# Telacebec Interferes with Virulence Lipid Biosynthesis Protein Expression and Sensitizes to Other Antibiotics

**DOI:** 10.3390/microorganisms11102469

**Published:** 2023-09-30

**Authors:** Zhiyu Zhou, Ruddy Wattiez, Patricia Constant, Hedia Marrakchi, Karine Soetaert, Vanessa Mathys, Véronique Fontaine, Sheng Zeng

**Affiliations:** 1 Microbiology, Bioorganic & Macromolecular Chemistry Research Unit, Faculté de Pharmacie, Université libre de Bruxelles (ULB), Boulevard du Triomphe, 1050 Brussels, Belgium; zhiyu.zhou@ulb.be; 2 Laboratory of Proteomics and Microbiology, Research Institute for Biosciences, University of Mons, Place du Parc 23, 7000 Mons, Belgium; 3 Institut de Pharmacologie et de Biologie Structurale (IPBS), Université de Toulouse, CNRS, Université Toulouse III—Paul Sabatier (UT3), 31077 Toulouse, France; 4 National Reference Laboratory “Mycobacterium”, Sciensano, 1180 Uccle, Belgium; 5 School of Nursing and Health, Nanfang College Guangzhou, Guangzhou 510970, China

**Keywords:** *Mycobacterium bovis* BCG, telacebec, vancomycin, PDIMs, virulence lipids

## Abstract

Tuberculosis (TB), caused by *Mycobacterium tuberculosis* (Mtb), remains a public health issue, particularly due to multi-drug-resistant Mtb. The bacillus is wrapped in a waxy envelope containing lipids acting as essential virulence factors, accounting for the natural antibiotic resistance of mycobacteria. Telacebec (previously known as Q203) is a promising new anti-TB agent inhibiting the cytochrome *bc*_1_ complex of a mycobacterial electron transport chain (ETC). Here, we show that the telacebec-challenged *M. bovis* BCG exhibited a reduced expression of proteins involved in the synthesis of phthiocerol dimycocerosates (PDIMs)/phenolic glycolipids (PGLs), lipid virulence factors associated with cell envelope impermeability. Consistently, telacebec, at concentrations lower than its MIC, downregulated the transcription of a PDIM/PGL-synthesizing operon, suggesting a metabolic vulnerability triggered by the drug. The drug was able to synergize on BCG with rifampicin or vancomycin, the latter being a drug exerting a marginal effect on PDIM-bearing bacilli. Telacebec at a concentration higher than its MIC had no detectable effect on cell wall PDIMs, as shown by TLC analysis, a finding potentially explained by the retaining of previously synthesized PDIMs due to the inhibition of growth. The study extends the potential of telacebec, demonstrating an effect on mycobacterial virulence lipids, allowing for the development of new anti-TB strategies.

## 1. Introduction

Tuberculosis (TB), caused by *Mycobacterium tuberculosis* (Mtb), remains the first leading cause of deaths from a single infectious agent. Among the 10.6 million people developing active TB in 2021, an estimated 450,000 patients suffered from multi-drug-resistant TB, which, by definition, is resistant to rifampicin and isoniazid, two of the core anti-TB drugs [1]. Drug-resistant TB remains an increasingly severe public threat as it is only curable with relatively limited effective drug options showing eventually severe side effects. Therefore, there is an urgent need to find more appropriate drug combinations, particularly based on novel anti-TB agents.

Mtb and the closely related vaccine strain *M. bovis* BCG (BCG) are obligate aerobic slow-growing mycobacteria producing their energy (i.e., ATP) predominantly through oxidative phosphorylation using the proton motive force generated in the electron transport chain (ETC) [2]. Mycobacterial ETC has received intensive attention, leading to the discovery of bedaquiline and telacebec (previously known as Q203). Telacebec, an imidazopyridine currently in clinical development, inhibits the cytochrome *bc*_1_:*aa*_3_ complex by binding to the menaquinol-binding site of the QcrB subunit, forcing the bacilli to use the less energetically efficient cytochrome *bd* oxidase [3,4,5,6]. This ETC rerouting renders the activity of telacebec only bacteriostatic [6,7]. Recent work thus focused on the identification of novel agents targeting mycobacterial cytochrome *bd* oxidase, with the aim of fully inhibiting the ETC, with some promising results obtained [6,8,9]. The continuous identification of other telacebec-based drug combinations will benefit from a thorough understanding of mycobacterial adaptations, in response to telacebec.

Mycobacterial species such as Mtb and BCG, similarly harboring a waxy cell envelope containing phthiocerol dimycocerosates (PDIMs) and structurally related phenolic glycolipids (PGLs), are inherently resistant to numerous antibiotics, including vancomycin, a glycopeptide antibiotic targeting the peptidoglycan biosynthesis [10,11,12]. PDIMs and PGLs are two surface-exposed methyl-branched lipids that are only present in pathogenic mycobacteria and BCG [11,13,14]. Genes required for their synthesis and transport are clustered in a ~50-kilobase fragment of Mtb/BCG chromosome containing several transcriptionally coupled operons, including the *fadD26*-*papA5* operon [15,16]. The *fadD26*-*papA5* operon, starting from the *fadD26* gene, encodes proteins involved in PDIM/PGL synthesis and translocation, including FadD26, the PpsA-E and DrrA-C proteins [16]. Mycobacterial mutants with disruptions in the PDIM/PGL biosynthetic or transport pathway exhibit significantly impaired virulence [17,18], demonstrating that PDIMs/PGLs are important virulence factors. PDIMs not only promote mycobacterial macrophage entry, but also prevent phagosomal acidification, permeabilize phagosomal membranes in infected macrophages, and prompt bacillary dissemination by facilitating macrophage necrosis [14,18,19,20]. Importantly, PDIMs and PGLs also contribute to the cell envelope integrity [21]. We previously reported that in BCG and Mtb, loss of the lipids is linked to an impaired biofilm phenotype and heightened susceptibility to multiple antibiotics, including rifampicin and vancomycin [11,12]. Subversion of Mtb virulence and intrinsic antibiotic resistance was indeed observed using inhibitors of the PDIM/PGL synthetic pathway [10,22]. Identification of other drugs that could directly or indirectly affect PDIM/PGL production could boost the discovery of more effective treatment options for managing drug-resistant TB.

The aim of the study was to further investigate the potential of telacebec, through the proteomic analysis of the telacebec-treated BCG, a suitable and biosafe model organism to study Mtb escape mechanisms and antibiotic susceptibility. The proteomic analysis revealed a potential effect of telacebec on the mycobacterial production of PDIMs/PGLs, and we further explored whether the drug could synergize with other antibiotics, e.g., vancomycin and rifampicin.

## 2. Materials and Methods

### 2.1. BCG Strains

BCG GL2, a strain issued from the BCG Pasteur strain, was described previously [12]. The BCG strain was maintained in 7H9 medium supplemented with 0.05% Tween 80 and 10% albumin-dextrose-catalase (ADC) enrichment (Becton, Dickinson and Company, Franklin Lakes, NJ, USA).

### 2.2. Construction of the pfadD26-luxAB Plasmid

The *E. coli*/mycobacteria shuttle pSMT1 plasmid, harboring *Vibrio harveyi luxA* and *luxB* genes under the transcriptional control of the Mtb *cpn60.2* promoter and the hygromycin B resistance gene [23], was digested by *Ale*I (14 bp upstream of P*_hsp60_*) and *Sna*BI (87 bp downstream of the *luxB* start codon) to remove the original promoter P*_hsp60_*, obtaining the vector. A 705 bp 5′-upstream region of *fadD26*, the first gene of the *fadD26*-*papA5* operon, was subjected to promoter prediction using BPROM (http://softberry.com, first accessed on 1 July 2018). Two inserts (A and B) were prepared for the subsequent In-Fusion Cloning. The insert A was the 705 bp upstream sequence before the first gene in the *fadD26*-*papA5* operon [15]. The insert B was the missing part (*luxA* plus 87 bp of *luxB*) of the *luxAB* gene after the enzymatic digestion of pSMT1. The inserts A and B were amplified using, respectively, BCG genomic DNA and pSMT1 as templates. The primer pairs used for the PCRs are described in Table S1. The vector, insert A, and insert B were ligated via homologous recombination using an In-Fusion HD Cloning Kit (Takara, Shiga, Japan). The p*fadD26*-*luxAB-*transformed *E. coli,* resistant to hygromycin, were identified by PCR and verified subsequently by sequencing.

### 2.3. The pfadD26-luxAB-Transformed BCG

Fresh culture of BCG, at an optical density at 600 nm (OD_600_) of 0.9, was harvested by centrifugation at 4000× *g* for 10 min. To obtain competent BCG, the bacterial pellet was washed 4 times with 50, 25, 10, and 5 mL ice-cold 10% (*v*/*v*) glycerol, before final resuspension in 1–2 mL ice-cold 10% glycerol. The competent BCG was transformed by electroporation. Briefly, 500–1000 ng of plasmids were mixed with 200 µL competent cells. The mixture was transferred to a 0.2 cm cuvette, followed by electroporation (EC_3_ set to 3 kV for 5.5–6 msec) using a Bio-Rad electroporator. After electroporation, bacteria were allowed to recover for 12 h at 37 °C in 7H9/0.05% Tween 80/10% ADC without antibiotics, before plating on 7H11 agar with 50 µg/mL hygromycin B.

### 2.4. Bioluminescence Detection

BCG harboring the p*fadD26-luxAB* plasmid was grown with 50 µg/mL hygromycin B to an OD_600_ of 0.4–0.6, followed by treatment with telacebec and measurement of LuxAB activity. Briefly, 100 µL of the freshly prepared substrate (18.8 µL decanal in 10 mL ethanol, adapted from [23]) was injected into 900 µL bacterial culture, and bioluminescence was measured using Lumat LB 9507 (Berthold) for 10 s.

### 2.5. Drug Susceptibility and Synergy Assessment

The BCG strain was grown at 37 °C in 7H9/0.05% Tween 80/10% ADC until OD_600_ reached 0.3–0.9. The macrodilution assay was performed in 12 mL screw-capped tubes containing 7H9 medium supplemented with 0.2% glycerol and 10%ADC, without Tween 80. Five hundred microliter inoculum diluted to reach an OD_600_ of 0.01 was added to 500 μL serial drug dilutions in 7H9/0.02% glycerol/10% ADC. Tubes were placed at 37 °C without shaking. Growth was recorded on the day when the growth of the drug-free control containing 1/100 inoculum became visible, in order to assess the minimal inhibitory concentration (MIC, being the lowest drug concentration inhibiting >99% of bacterial growth) [10]. This experiment was performed three times to ascertain the reproducibility of the MIC assay. The fractional inhibitory concentration index (FICI) of telacebec in combination with vancomycin or rifampicin was calculated according to the formula FICI = FIC_a_ + FIC_b_ = MIC_ab_/MIC_a_ + MIC_ba_/MIC_b_. The MIC of telacebec (MIC_a_) and vancomycin/rifampicin alone (MIC_b_), the MIC of telacebec in combination with a fixed vancomycin/rifampicin concentration (MIC_ab_), and inversely the MIC of vancomycin/rifampicin in combination with a fixed telacebec concentration (MIC_ba_) were obtained. In agreement with the checkerboard method, synergy was reached when FICI was under 0.5 [24].

### 2.6. Protein Extraction

The biomass of the heat-inactivated bacteria was prepared as previously described [25]. Bacteria were first lysed in 6 M guanidine HCl, 50 mM K_2_HPO_4_/KH_2_PO_4_ (pH 8.5), followed by ultrasonication (3 × 10 s, 20% amplitude; U50 IKA Technik). Fifty milligrams of the extracted proteins were reduced and alkylated. The proteins were recovered by acetone precipitation and digested with 0.005% (*w*/*v*) trypsin (Promega V5111, Madison, WI, USA) in 25 mM NH_4_HCO_3_ (pH 8.5). The trypsin treatment was stopped by 0.1% formic acid (*v*/*v*).

### 2.7. MS Analysis, SWATH Acquisition, and Data Interpretation

Prior to mass spectrometry (MS) analysis, reverse-phase chromatography was used to separate the extracts. The reverse-phase column (15 cm in length, 75 mm in diameter, flow 300 nL/min; PepMap C18, Thermo Scientific Dionex, Waltham, MA, USA) was equilibrated with 4% (*v*/*v*) acetonitrile for 20 min, and peptide elution was carried out over an acetonitrile gradient from 4% to 35% (*v*/*v*) for 120 min. The separated peptides were then analyzed online by a TripleTOF 5600 mass spectrometer (AB Sciex, Framingham, MA, USA). Peptide spectra were acquired in data-dependent (DDA) and data-independent (DIA) acquisition modes. The MS/MS library was acquired in the DDA mode and analyzed by the ProteinPilot software (version 4.5, AB Sciex, USA) using the algorithm Paragon (version 4.5.0.0, AB Sciex, USA). Briefly, the trypsin was chosen as the cleavage specificity and alkylation (C) set to iodoacetamide, carbamidomethylation as fixed modifications, oxidation (M) and deamination (N, Q) as variable modifications were set. All biological modifications and amino acid substitutions were considered, and a thorough ID search was applied with peptide confidence set at 0.99. The raw spectral data obtained served as the input for ProteinPilot against the *Mycobacterium bovis* UniProt database. For the database search, the cut-off peptide confidence limit was set at 95%. ProteinPilot provided a global false discovery rate of 1% and a local false discovery rate of 5%. Accumulation time was set to 0.1 s for the MS1 scan and 65 ms for the MS2 scan, with the total cycle time being approximately 3.8 s.

For the SWATH analysis (DIA, AB Sciex), 32 incremental steps defined as windows of 25 *m*/*z* containing 1 *m*/*z* for the overlap of the window were passed over the full mass range (400–1250 *m*/*z*).

The peak intensity method was used for the quantitation of peptides. The ion chromatogram of the top six fragmented peptides was extracted, and their area was integrated over 15 min on six transitions. The tolerance was set at 100 ppm. The SWATH data were processed with the PeakView software (version 2.1.0.11041, AB Sciex, USA). The retention time was calculated manually from a group of 15 selected peptides with retention time in the range of 20–100 min. MarkerView Software (version 1.2.1, AB Sciex, USA) was used for the analysis of the relative abundance of the peptides. For all experiments, proteins identified with one peptide were rejected for interpretation. Only protein hits with a significant difference (*p* ˂ 0.05) and a fold change of ˂0.83 or ˃1.2 were further analyzed [26,27]. Protein identities could be found using the UniProt entry number at http://www.uniprot.org/. Protein function annotations and categories were based on UniProt (http://www.uniprot.org/, first accessed on 10 September 2022), Mycobrowser (https://mycobrowser.epfl.ch/, accessed on 12 September 2022), and/or the NCBI Conserved Domain Search tool (https://www.ncbi.nlm.nih.gov/Structure/cdd/cdd.shtml, accessed on 10 September 2022). In addition, NCBI BLAST (https://blast.ncbi.nlm.nih.gov/Blast.cgi?PAGE=Proteins, accessed on 12 September 2022) was applied to find homology proteins in Mtb for the identified BCG proteins.

### 2.8. Lipid Analysis

Bacteria were treated or not with telacebec at 50 nM (10 × MIC) for 2 days before being heat-inactivated. Lipids were then extracted twice with CHCl_3_/CH_3_OH (1:2 *v*/*v*), then (2:1 *v*/*v*), concentrated under vacuum, washed two times with water, and dried. The total lipid extracts were then solubilized in CHCl_3_ at 20 mg/mL. The DIM content of each extract (25 µL) was analyzed by thin-layer chromatography (TLC) on silica gel G 60 plates using petroleum ether/diethyl ether (9:1 *v*/*v*) as the eluent. Lipid compounds were visualized by spraying the plates with 10% phosphomolybdic acid in ethanol and heating. DIMs purified from Mtb were run as the control.

### 2.9. Statistical Analysis

The unpaired *t*-test was applied for comparisons between two groups (for the proteomic analysis). A *p*-value of ˂0.05 was considered statistically significant. The graphs were prepared with OriginPro 8 (OriginLab Corporation, Northampton, MA, USA) or with GraphPad Prism version 9.5.1 for Windows (GraphPad Software, San Diego, CA, USA).

## 3. Results

### 3.1. Decreased Abundance of Proteins Required for PDIM/PGL Synthesis by Telacebec

**Figure 1:**
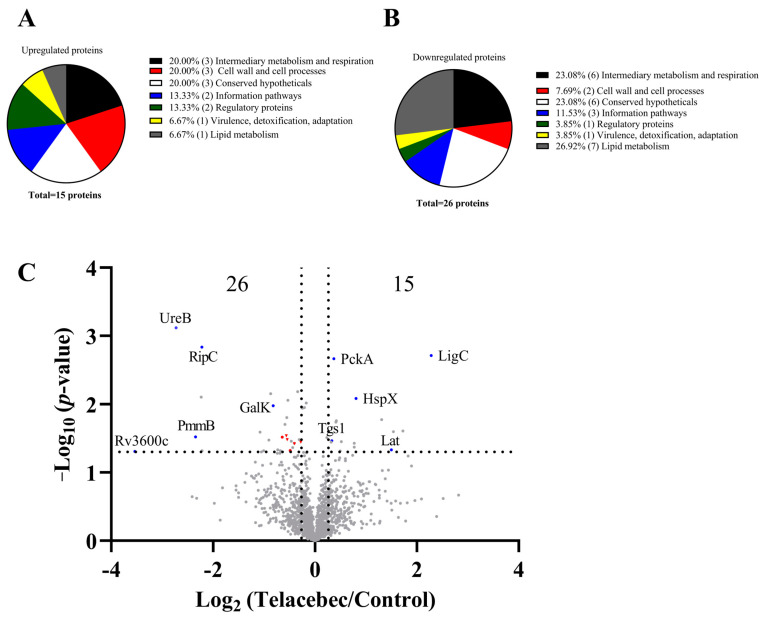
Proteomic profiling of BCG treated with 10 nM telacebec for 7 h. (**A**,**B**) Functional category analysis of the upregulated (**A**) and downregulated (**B**) proteins was performed based on Mycobrowser (https://mycobrowser.epfl.ch/, first accessed on 12 September 2022). For each classification, the number of proteins (in bracket) and the percentages are shown. (**C**) Volcano plot showing downregulated (upper left) and upregulated (upper right) proteins in the treated bacilli as compared with the untreated control. Each dot/triangle (in grey, red or blue color) indicates a different protein. Names of particular proteins are given (see blue dots). Proteins required for lipid metabolism are emphasized by red dots/triangles, with those involved in PDIM biosynthesis indicated by triangles. The dot line parallel to the *x*-axis indicates a y value of 1.301 (i.e., −log10·0.05). The two dot lines parallel to the *y*-axis indicate x values of −0.263 (i.e., log2·0.833) and 0.263 (i.e., log2·1.2), respectively.

We treated BCG with 10 nM telacebec (2 × MIC), showing only a bacteriostatic effect [7]. The proteomic analysis of the untreated and telacebec-treated BCG (at an early time point, 7 h) allowed for highlighting that 15 and 26 proteins were upregulated and downregulated, respectively, by telacebec (Figure 1). Six proteins categorized in the “intermediary metabolism and respiration” group were among the downregulated proteins (e.g., UreB (0.15-fold) and GalK (0.56-fold)), while 3 were found in the upregulated proteins (e.g., PckA (1.3-fold) and Lat (2.8-fold)) (Figure 1A–C). Among the upregulated proteins, at least 5 play a role in virulence, including EspD (1.7-fold), HspX (1.7-fold), PhoR (1.25-fold), MprA (1.4-fold), and Lat (2.8-fold) (Table 1). Notably, among the upregulated proteins, only 1 was involved in “lipid metabolism” (i.e., Tgs1, 1.3-fold). In contrast, 7 proteins grouped in “lipid metabolism” were identified in the downregulated proteins (Figure 1A,B), suggesting a general downregulation of lipid metabolism by telacebec. Among the proteins involved in “lipid metabolism”, 6 are associated with lipid biosynthesis (i.e., Fas, FadD22, AccD6, FadD26, FadD29, and PpsB; see red dots/triangles in Figure 1C), especially in PDIM/PGL biosynthesis (i.e., FadD22, FadD26, FadD29, and PpsB, all downregulated by telacebec; see red triangles in Figure 1C) (Table 1). FadD26 and PpsB are expressed from the fadD26-papA5 operon, and FadD22/FadD29 from another. Other proteins expressed from the operons were not identified in our proteomic assay. Hence, telacebec may interfere with the production of PDIMs/PGLs, important virulence factors associated with the cell envelope impermeability of the pathogenic slow-growing mycobacteria.

### 3.2. Telacebec Decreased the fadD26 Promoter Activity

**Figure 2:**
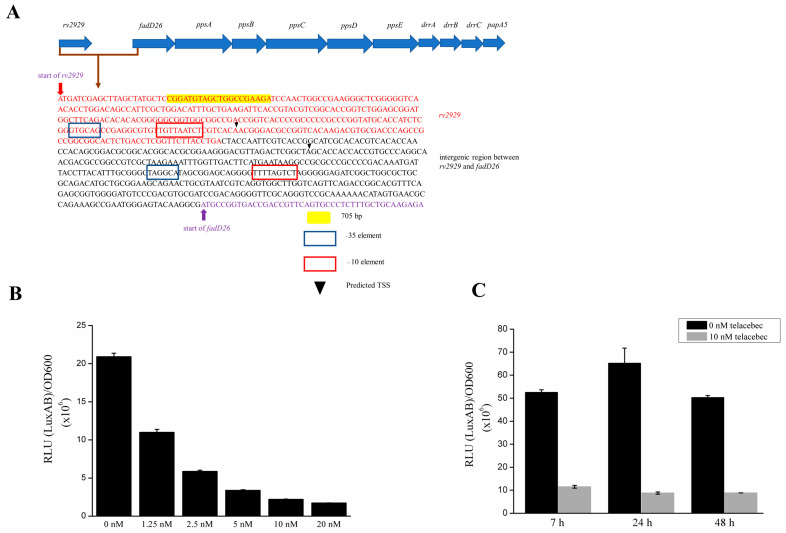
Telacebec decreased the *fadD26* promoter activity. (**A**) Characterization of the *fadD26* promoter region. The annealing site for the forward primer used to amplify the 705 bp upstream of the *fadD26* gene is colored (yellow). The reverse primer annealed just before the start of *fadD26*. The two predicated −35 and −10 elements are boxed. TSS: transcriptional start site. The two TSSs (indicated by arrows) were described previously [28]. Gene names were based on Mtb H37Rv. (**B**) Exponential p*fadD26*-luxAB-transformed BCG cultures were treated with indicated concentrations of telacebec for 24 h, followed by RLU measurement. The data were normalized by dividing by OD_600_. (**C**) Exponential p*fadD26*-luxAB-transformed BCG cultures were treated with 10 nM telacebec for the indicated time points, followed by RLU measurement. The data were normalized by dividing by OD_600_. The *fadD26* promoter activity was already decreased after 7 h of telacebec treatment.

We further assessed the expression of the faD26-papA5 operon, as representative PDIM/PGL-synthesizing gene loci. The transcriptional activity of the first promoter of this operon, upstream to the fadD26 gene, was investigated using a luciferase reporter system (Figure 2A) [15]. We constructed the pfadD26-luxAB plasmid containing the luxAB luciferase genes from Vibrio harveyi in the pSMT1 vector [23]. A 705 bp 5′-upstream region of the fadD26 gene, predicted to harbor 2 putative promoter sites using the BPROM software (http://softberry.com, accessed on 1 July 2018) and reported to contain 2 transcriptional start sites (TSS) (Figure 2A), was inserted in the pSMT1 vector to replace the original mycobacterial cpn60.2 promoter to control the luxAB transcription [23,28]. The luciferase activity obtained in the pfadD26-luxAB-transformed BCG showed high RLU values, demonstrating a strong promoter activity (Figure 2B). This activity was strongly reduced after 24 h telacebec treatment, depending on the telacebec concentration, achieving a 2- to 12-fold reduction. Notably, 1.25 nM telacebec (1/4 of the MIC) already decreased the promoter activity (Figure 2B), suggesting that the effect on PDIMs/PGLs occurs before the inhibition of bacillary growth. This effect was observed after 7 h treatment (Figure 2C), in agreement with our proteomic data showing a decreased level of proteins encoded in the operon.

### 3.3. Telacebec Synergized with Vancomycin and Rifampicin

We previously showed that PDIMs are required to reduce vancomycin susceptibility in both Mtb and BCG [12]. Moreover, loss of PDIMs also increases mycobacterial susceptibility to other antibiotics, such as rifampicin [12,29]. The reduced level of proteins required for PDIM/PGL synthesis (Table 1) and the decreased fadD26 promoter activity (Figure 2) following telacebec treatment could suggest a potentiating effect of telacebec on other antibiotic susceptibility. This was investigated by the macrodilution method on BCG. In the presence of vancomycin (5 μg/mL, far below the MIC), the MIC of telacebec fell from 5 nM (without vancomycin) to 1.25 nM, while in the presence of a fixed amount of telacebec (1.25 nM), the MIC of vancomycin fell from >200 μg/mL (without telacebec) to 5 μg/mL (Table 2). Likewise, in the presence of rifampicin (0.0625 μg/mL), the MIC of telacebec fell from 5 nM (alone) to 1.25 nM, while in the presence of a fixed amount of 1.25 nM telacebec, the MIC of rifampicin fell from 0.5 μg/mL (without telacebec) to 0.0625 μg/mL (Table 3). The calculation of FICI, showing values below 0.5 for both combinations, telacebec/vancomycin and telacebec/rifampicin, demonstrated that telacebec synergized with vancomycin and with rifampicin.

**Figure 3:**
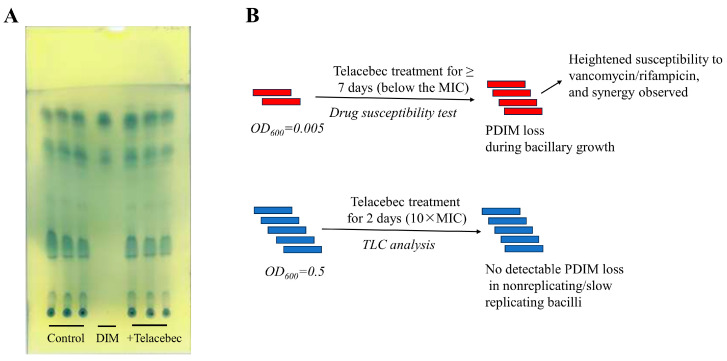
TLC analysis for the detection of PDIM amount in telacebec-treated BCG. (**A**) BCG was treated or not with telacebec (50 nM) for 2 days, followed by TLC analysis of the extracted lipids. DIM: DIMs purified from Mtb were run as a control. The experiment was performed twice, each in triplicate and one representative data shown. (**B**) The red/blue bars mark individual mycobacterial cells, and the number of bars indicates bacillary growth (increasing number for red bars) or not (same number for blue bars). In the synergy assay, the concentration of telacebec is 1/4 of the MIC (i.e., 1.25 nM), failing to inhibit the bacillary growth but already decreasing the *fadD26* promoter activity. In contrast, in the TLC analysis, a much higher growth-inhibiting concentration of telacebec was used.

To investigate whether the synergy could be due to a potential effect of telacebec on PDIM production, TLC analysis was performed. Relative to control, the telacebec (10 × MIC)-treated BCG did not show a detectable decrease in PDIM amount (Figure 3A). However, based on the data, the effect of telacebec on PDIM production could not be negated as this concentration of telacebec could expectedly inhibit the growth of the bacilli, potentially leading to the retaining of previously synthesized cell wall PDIMs (Figure 3B).

## 4. Discussion

### 4.1. Effect of Telacebec on PDIMs: A Point of Metabolic Vulnerability

Telacebec, currently in clinical trials, inhibits the cytochrome *bc*_1_/*aa*_3_ route, leading to respiratory rerouting through the alternative cytochrome *bd* oxidase pathway [30]. This rerouting results in a rapid and significant decrease in cellular ATPs [8,30]. Mycobacterial exposure to bedaquiline, likewise inhibiting the ATP production in the respiratory chain, could trigger multiple metabolic vulnerabilities (e.g., glutamine synthetase) that can be leveraged for designing synergistic drug combinations [31,32]. However, a picture of metabolic vulnerabilities as a consequence of telacebec treatment has yet to be uncovered. Here, we show that telacebec interferes with the mycobacterial production of virulence lipids, PDIMs, allowing for potentiating vancomycin and rifampicin.

The over-representation of downregulated proteins involved in lipid metabolism in the telacebec-treated bacilli could be linked to the fact that the drug markedly decreases ATPs, indispensable for the energy-consuming lipid biosynthetic pathway [7,33]. Lupien et al. observed that in Mtb treated with another cytochrome *bc*_1_ inhibitor, genes involved in lipid metabolism were over-represented in the downregulated gene set [34], suggesting a similar phenotype in the pathogenic mycobacteria. Proteins (e.g., FadD22, FadD26, FadD29, and PpsB) required for the PDIM/PGL biosynthesis, as well as the transcriptional activity of the *faD26*-*papA5* operon, were downregulated quickly by telacebec, even at concentrations lower than the MIC. Hence, the effect of telacebec on PDIMs/PGLs seems to occur before the drug-induced growth stasis. In agreement, the nutrient-starved Mtb, producing lower ATPs, also downregulated the expression of genes required for the PDIM/PGL synthesis [35,36]. Moreover, the bedaquiline-treated Mtb also downregulated the expression of proteins involved in lipid metabolism, mostly in the biosynthesis of mycolic acids and PDIMs/PGLs [37]. Hence, the decreased biosynthesis of PDIMs/PGLs, part of a lipid reprogramming program, seems to be a general adaptive strategy employed by the stressed bacilli producing prominently reduced ATPs. How the PDIM/PGL biosynthesis could quickly respond to and coordinate with the extracellular stresses remains to be investigated.

PDIMs and PGLs, the two structurally related methyl-branched lipid esters, are noncovalently associated with the outer membrane of the pathogenic Mtb, of *M. marinum*, of *M. bovis,* and of BCG [12,14,15,38]. The two lipids, particularly PDIMs, contribute to cell envelope impermeability required for mycobacterial natural antibiotic resistance, in both the attenuated BCG and the pathogenic Mtb [12,15,39]. In the present study, the vaccine strain, BCG, was used for biosafety reasons. BCG and Mtb, sharing ~98% identity of their genomes [40], respond to stresses, generally, in a similar pattern. For instance, both organisms quickly and significantly decreased cellular ATP production and reduced their growth rates in response to telacebec [7,8]. To the best of our knowledge, the transcriptomic or proteomic data of the telacebec-treated mycobacteria (including Mtb) have not been reported before. It may be questioned whether the finding that telacebec could interfere with BCG PDIMs can be extended to Mtb. We note that treatment of Mtb H37Ra with TB47, another cytochrome *bc*_1_ inhibitor [8], downregulated the expression of proteins involved in PDIM biosynthesis (unpublished observations). Furthermore, the ATP-depleting bedaquiline also decreases the Mtb expression of proteins involved in the synthesis of lipids, particularly PDIMs [37]. We previously reported that like BCG, Mtb deficient for PDIMs is hypersensitive to vancomycin [12]. Hence, it is reasonable to extrapolate that telacebec could also interfere with PDIM production in Mtb and hence potentiate rifampicin and vancomycin in the pathogenic bacilli.

More importantly, PDIMs/PGLs may exert functions far beyond maintaining cell envelope integrity, as demonstrated by their involvement in modulating the bacillary life cycle and in mycobacterial virulence [14,19,20,41]. Considering this, the in vivo anti-TB efficacy of telacebec may be even more potent. Indeed, a phase 2 clinical trial found that telacebec shows an early bactericidal activity when used to treat newly diagnosed pulmonary TB patients, as indicated by the gradual reductions in bacterial sputum load [42]. Hence, the PDIM/PGL downregulation, potentially responding to lower ATPs, may be considered a point of telacebec-induced metabolic vulnerabilities, which could be further targeted by the host immune responses and by other therapeutic agents. Previous work has revealed several points of metabolic vulnerability induced by the ETC-targeting bedaquiline, such as glycolytic vulnerability and glutamine synthetase vulnerability [31,32], resulting in the formation of more effective bedaquiline-based drug combinations.

Unexpectedly, the TLC analysis did not show a significant loss of cell wall PDIMs in telacebec-treated bacilli, a finding seemingly contradictory to our data obtained in the proteomic and promoter analysis. This could be explained by a potential effect of growth on previously synthesized PDIMs (Figure 3B). In the drug synergy assay, the telacebec concentration is 1/4 of the MIC (i.e., 1.25 nM), a concentration failing to inhibit the bacillary growth but already decreasing the *fadD26* promoter activity (Figure 2). Hence, a gradual loss of cell wall PDIMs can be expected as a result of active growth, eventually leading to the heightened susceptibility to vancomycin/rifampicin. In stark contrast, a significantly higher concentration of telacebec used in the TLC analysis is expected to inhibit the bacterial growth, potentially leading to the retaining of cell wall PDIMs that were synthesized before the addition of telacebec (Figure 3B). Detection of newly synthesized PDIMs, such as by the addition of a radio-labelled precursor (e.g., 14C propionate) just after the addition of telacebec, may provide a definitive answer.

### 4.2. Other Proteomic Signatures by Telacebec

Proteomic analysis also identified changes of several other proteins. For instance, the analysis identified proteins involved in bacterial growth and cell division, such as the ZipA-like protein Rv3835 (0.2-fold) potentially involved in the septum formation [43] and the Rv2190c protein (RipC, 0.2-fold), a peptidoglycan peptidase required for *Mtb* active growth [44,45]. In addition, two proteins expressed in the dormancy regulon (also known as the DosR regulon), i.e., Tgs1 (1.26-fold) and HspX (1.75-fold), were induced by telacebec (Figure 1C). The DosR regulon, comprising nearly 50 genes, is activated in response to multiple stresses, such as hypoxia, and its activation contributes to mycobacterial survival and antibiotic tolerance [46,47,48]. Bedaquiline treatment also activates the DosR regulon in Mtb [37], suggesting a similar mycobacterial adaptation strategy in response to these ETC-targeting agents.

The proteomic analysis also gave additional interesting information. Rv3600c, a putative pantothenate kinase involved in the coenzyme A (CoA) biosynthetic pathway [49], was the most downregulated (0.086-fold) by telacebec (Figure 1C). This protein is expressed from a putative operon containing genes coding for PanC and PanD, two proteins participating in the biosynthesis of pantothenate, a CoA precursor [50]. Knowing that the cofactor CoA or pantothenate is required for lipid biosynthesis by polyketide synthesis proteins [51], such as the Pps proteins involved in PDIM/PGL biosynthesis, this observation could further highlight the impact of telacebec on PDIM/PGL biosynthesis. Intriguingly, the downregulation of Rv3600c was not previously identified by other cytochrome *bc*_1_ inhibitors [34,52].

### 4.3. Conclusions

In conclusion, telacebec interferes with virulence lipid (PDIMs/PGLs) biosynthesis protein expression, considered as a point of the drug-induced metabolic vulnerabilities that could be further exploited for, at least, antibiotic potentiation. The finding expands the potential of telacebec, likely also other ATP-interfering agents, and opens up an avenue for formulating telacebec-based rational drug combinations. For instance, the synergistic combination of telacebec and vancomycin (poor oral bioavailability) could be further assessed for its anti-TB efficacy in animal TB models, followed by formulating dry powders for inhalation or by employing efficient delivery systems to achieve optimal pulmonary administration of the drug combination, a potential strategy for treating drug-resistant pulmonary TB patients.

## Figures and Tables

**Table 1:** Proteomic profiling of BCG treated with 10 nM telacebec for 7 h.

	BCG Homology Proteins in H37Rv	Name	Description	*p*-Value	Fold Change (Telacebec/Control)
Cell wall biosynthesis and cell process	Rv2950c	FadD29	Long-chain-fatty-acid-AMP ligase	0.02887	0.677371423
	Rv2247	AccD6	Acetyl-/propionyl-CoA carboxylase subunit beta	0.03024	0.641045938
	Rv2930	FadD26	Fatty-acid-AMP ligase	0.03297	0.685370986
	Rv3130c	Tgs1	Diacylglycerol O-acyltransferase	0.03431	1.258329713
	Rv2524c	Fas	Fatty acid synthase	0.03723	0.754112466
	Rv2932	PpsB	Phthiocerol synthesis polyketide synthase type I	0.03774	0.755835038
	Rv2190c	RipC	Peptidoglycan peptidase	0.00146	0.214892227
	Rv3484	CpsA2	Polysaccharide transfer to peptidoglycan	0.00653	0.789836749
	Rv3835	NA	Hypothetical protein in the cell wall	0.00786	0.212690355
	Rv3915	NA	Peptidoglycan hydrolase	0.01965	0.664403424
Metabolic pathways	Rv0211	PckA	Phosphoenolpyruvate carboxykinase	0.00217	1.294218998
	Rv0620	GalK	Galactokinase	0.01055	0.566918099
	Rv3308	PmmB	Phosphomannomutase; converts D-mannose 1-phosphate to D-mannose 6-phosphate	0.0301	0.196754059
	Rv3600c	NA	Type III pantothenate kinase catalyzing the phosphorylation of pantothenate, the first step in CoA biosynthesis	0.04899	0.086255116
	Rv1589	BioB	Biotin synthetase	0.01568	0.681640846
	Rv0991c	NA	Redox-regulated molecular chaperone	0.00875	0.693003332
	Rv0758	PhoR	Two-component system response sensor kinase	0.0358	1.247705251
Virulence	Rv0485	NA	Transcriptional regulator of pe13 and ppe18 gene pair	0.04811	0.497470112
	Rv0981	MprA	Two-component response regulator	0.04824	1.407815183
	Rv2031c	HspX	Alpha-crystallin	0.00824	1.749009088
	Rv0726c	NA	Possible methyltransferase associated with isoniazid resistance	0.00699	0.547160643
	Rv3867	EspH	ESX-1 secretion-associated protein	0.04805	0.625076945
	Rv3614c	EspD	ESX-1 secretion-associated protein	0.03731	1.704031265
DNA/RNA metabolism	Rv1629	PolA	DNA polymerase I	0.01742	1.338907985
	Rv1641	InfC	Initiation factor IF-3	0.03486	0.725560396
	Rv3731	LigC	DNA ligase C	0.00194	4.862120018
Others	Rv1287	NA	HTH-type transcriptional regulator	0.02526	2.906914209
	Rv3200c	NA	Transmembrane cation transporter	0.01681	2.480604421
	Rv1072	NA	Transmembrane protein	0.0353	1.432802446
	Rv1849	UreB	Urease subunit beta	0.00076	0.150994385
	Rv3011c	GatA	Glutamyl-tRNA amidotransferase subunit A	0.04319	0.738761056
	Rv3290c	Lat	L-lysine-epsilon aminotransferase; catalytic activity: L-lysine + 2-oxoglutarate = 2-aminoadipate 6-semialdehyde + L-glutamate	0.04644	2.83040088

NA: not applicable.

**Table 2:** Synergy between telacebec and vancomycin.

Treatment	MIC/FIC/FICI	
	Vancomycin (μg/mL)	Telacebec (nM)
Alone	>200/-/-	5/-/-
+Telacebec (nM)	5/<0.025/<0.275	-/-/-
+Vancomycin (μg/mL)	-/-/-	1.25/<0.25/<0.275

**Table 3:** Synergy between telacebec and rifampicin.

Treatment	MIC/FIC/FICI	
	Rifampicin (μg/mL)	Telacebec (nM)
Alone	0.5/-/-	5/-/-
+Telacebec (nM)	0.0625/0.125/0.375	-/-/-
+Rifampicin (μg/mL)	-/-/-	1.25/0.25/0.375

## Data Availability

Data supporting the reported results are available from the corresponding author upon request.

## Supplementary Materials

The following supporting information can be downloaded at https://www.mdpi.com/article/10.3390/microorganisms11102469/s1, Table S1: Primers used in the study; Excel file: Proteomic profiling of BCG treated with 10 nM telacebec for 7 h.
