# Supplementary material for: Telacebec Interferes with Virulence Lipid Biosynthesis Protein Expression and Sensitizes to Other Antibiotics
Source: Microorganisms. 2023 Sep 30;11(10):2469. doi: 10.3390/microorganisms11102469 (PMC10609169; doi:10.3390/microorganisms11102469)
Supplement: Supplementary file 1 [file microorganisms-11-02469-s001.zip › Table S1.pdf]

Primers used in the study

To amplify	Primer pairs	Sequence
The 705 bp upstream of *fadD26* (insert A)	Forward	5′-ATCTGGCTCGCACCGCGGATGTAGCTGGCCGAAGA-3′
	Reverse	5′-AATTGTCTTGGCCATCGCCTTGTACTCCCATTCG-3′
LuxA plus 87 bp of *luxB* (insert B)	Forward	5′-ATGGCCAAGACAATTGCGGATCCA-3′
	Reverse	5′-CTTCAACTGATCTACGTAATGTGCGG-3′
